# Corrosion Resistance and Wear Behavior of Ni60/TiC and NbC Composite Coatings Prepared by Laser Cladding

**DOI:** 10.3390/ma18112459

**Published:** 2025-05-24

**Authors:** Qiang Zhan, Fangyan Luo, Jiang Huang, Zhanshan Wang, Bin Ma, Chengpu Liu

**Affiliations:** 1 Institute of Precision Optical Engineering, School of Physics Science and Engineering, Tongji University, Shanghai 200092, China; wangzs@tongji.edu.cn (Z.W.); mabin@tongji.edu.cn (B.M.); 2 State Key Laboratory of Ultra-Intense Laser Science and Technology, Shanghai Institute of Optics and Fine Mechanics, Chinese Academy of Sciences, Shanghai 201800, China; chpliu@siom.ac.cn; 3 Center of Materials Science and Optoelectronics Engineering, University of Chinese Academy of Sciences, Beijing 100049, China; 4 School of Electronics and Information Engineering, Guangdong Ocean University, Zhanjiang 524088, China; 18922863861@163.com (F.L.); huangjiang@gdou.edu.cn (J.H.)

**Keywords:** Ni60, TiC, NbC, corrosion resistance, wear behavior, laser cladding

## Abstract

This research delves into the corrosion resistance and wear behavior of Ni60-based composite coatings strengthened by TiC and NbC particles, which are produced by laser cladding. Three distinct coatings were prepared: S1 (Ni60 + 20%TiC), S2 (Ni60 + 10%TiC + 10%NbC), and S3 (Ni60 + 20%NbC). Microstructural characterization revealed that the addition of TiC and NbC altered phase composition, inducing lattice distortion and promoting the formation of carbides such as Cr_7_C_3_, Ni_3_C, and Nb_2_C. The S2 coating exhibited the highest average microhardness (1045 HV) due to synergistic grain refinement and homogeneous carbide dispersion. Wear resistance followed the order S2 > S3 > S1, attributed to the optimized balance of hard-phase distribution and reduced abrasive wear. Electrochemical tests in 3.5 wt% NaCl solution demonstrated superior corrosion resistance for S3, characterized by the lowest corrosion current density (1.732 × 10^−6^ A/cm^2^) and a stable passivation film, facilitated by NbC-induced oxide formation. While S2 achieved peak mechanical performance, S3 excelled in corrosion resistance, highlighting the trade-off between carbide reinforcement and electrochemical stability. This work underscores the potential of tailoring dual-carbide systems in Ni60 coatings to enhance durability in harsh environments.

## 1. Introduction

As a novel surface strengthening technology in the field of additive manufacturing [1,2,3,4,5], laser cladding (LC) has become an innovative approach in the field of material processing. The technology boasts notable strengths like minimal dilution, robust metallurgical connection to the substrate, and no environmental contamination. By leveraging LC, diverse metal powders can be melted and deposited on the surfaces of critical components. This procedure can generate high-performance coatings, bestowing the substrate with superior properties such as exceptional wear resistance, remarkable corrosion resistance, and outstanding high-temperature oxidation resistance [6,7,8,9,10]. Thanks to these prominent advantageous features, laser cladding has been widely applied in numerous industries, including aerospace, petrochemical, and transportation [11,12,13].

However, under harsh working conditions, the coatings made solely from LC pure self-fluxing alloy powder materials (such as Ni, Co, and Fe-based alloys) are unable to satisfy actual usage demands. Thus, numerous researchers have endeavored to further enhance the performance of the coatings by adding ceramic particles to the self-fluxing powders [14,15,16,17]. For instance, Ramasobane et al. [18] employed laser cladding to produce a TiC/Ti-6Al-4V cladding layer. The volume fraction of this layer was 3.85%, and it was applied to the surface of Ti-6Al-4V alloy. The average hardness was around 457.59 ± 39.73 HV0.3. This technique greatly improved the surface microhardness of the titanium alloy. Additionally, it also improved the wear resistance. Ghorbani et al. [19] created a TiC/Ti cladding layer. The layer was in situ synthesized on the Ti-6Al-4V alloy. They discovered that the microhardness of the cladding layer rose as the TiC content increased. Al-Sayed et al. [20,21] prepared a powder mixture onto the surface of the Ti-6Al-4V alloy by LC, with a mass ratio of 60% WC and 40% NiCrBSi. The average hardness of the cladding layer was three times higher than that of the substrate. Moreover, its wear resistance was significantly improved. Likewise, Elshaer et al. [22] applied the direct energy deposition technique. They deposited a cladding on the TC21 titanium alloy, which was composed of 40% Stellite-6 and 60% WC. They found that the cladding’s microhardness was three times higher than that of the substrate. Liu et al. [23] created a coating on the TC4 surface, with a mass ratio of 50% WC and 50% Ni60. In comparison to the substrate, the average hardness became twice as high, the friction coefficient (COF) dropped by around 80%, and the wear resistance was remarkably improved. Xia et al. [24] created Ni60A composite cladding layers on the TC4 alloy surface by LC technology. The layers contained various mass fractions of TiC, namely 0%, 2%, 4%, and 6%. In comparison with the substrate, the layer with 4% TiC had double the average hardness, and the friction coefficient was reduced by roughly 25%. Moreover, the wear resistance was improved. Li et al. [25] discovered that adding niobium carbide (NbC) particles to the AlCoCrFeNi high-entropy alloy (HEA) was beneficial. It could effectively enhance the hardness and wear resistance. Specifically, the hardness escalated from 200 Vickers hardness (HV) to 525 HV, while the wear amount dropped by around 37% from 1.66 mg. Jiang et al. [26], during their research, put NbC particles into the AlCoCrFeNi2.1 HEA for the purpose of enhancing its hardness and wear resistance. The research results showed that as the content of NbC increased from x = 0 to x = 1.0, the average friction coefficient of AlCoCrFeNi2.1- xNbC dropped from 0.59 to 0.42. Meanwhile, the wear rate dropped as well. It went from 1.5 × 10^−5^ mm^3^N^−1^ m^−1^ to 2.4 × 10^−6^ mm^3^N^−1^ m^−1^.

In recent research, Fan et al. [27] prepared a TC4/WC + TiC coating through laser cladding (LC) and studied the competitive mechanism of TiC and WC in improving the properties of the TC4 coating. The average microhardness of the 20 wt% TiC cladding layer was determined. It equaled 92% of the 20 wt% WC cladding layer. Nevertheless, the friction and wear rate of the 20 wt% TiC cladding layer was merely 41% of that of the 20 wt% WC. In addition, the electrochemical corrosion rate of the 20 wt% TiC cladding layer was also measured. It was just 20% of the latter. H.F. Zhang et al. [28] reported that the in situ synthesized NbC-reinforced iron-based composite coating was more resistant to corrosion in sodium chloride (NaCl) solution. H. Wu et al. [29] found that a highly ordered, dense and defect-free protective passivation film was formed on the surface of the FeNiCoCr + 10% (mass fraction) NbC cladding layer, which significantly improved its corrosion resistance.

Luara da Costa Morais et al. [30] investigated the effect of TiC addition on carbide particle growth in NbC-Ni hardmetals by powder metallurgy. They found that TiC addition improved hardness and toughness, with the highest values achieved in the 7% TiC-NbC-12% Ni composition (by mass). Guo-Ye Jiang et al. [31] studied the effects of laser power on the microstructure, microhardness, and friction properties of TiC-NbC coatings on stainless steel surfaces. They observed in situ formation of reinforcing phases such as (Ti,Nb)C and CrB during laser cladding, which positively influenced the microhardness of the coatings. Therefore, in this research work, a Ni60/NbC + TiC coating was prepared by using LC, and the influences of NbC and TiC, as carbide reinforcement phases, on the phase composition of the cladding layer were examined and contrasted in depth. The study detailed the precipitation behavior of the two carbides within the cladding layer. The characteristics were described. The morphology was analyzed. And the distribution was covered. In addition, this paper systematically studied the influence of the content of the reinforcement phase, which was exerted on the microhardness, wear resistance, and corrosion resistance of the cladding layer. It offers crucial insights for enhancing the functionality and properties of the Ni60/TiC and NbC alloy coatings fabricated through LC technology.

## 2. Experimental Procedures

**Figure 1:**
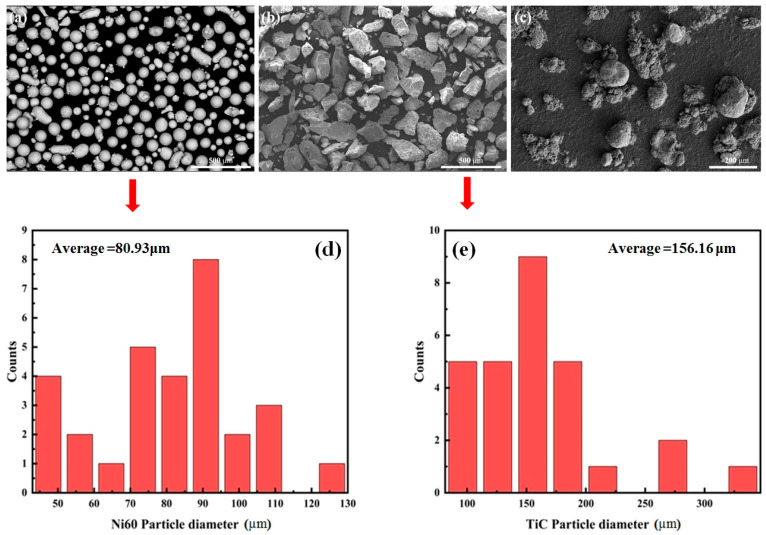
SEM images of the powders: (**a**) Ni60; (**b**) TiC; (**c**) NbC. Particle size distribution: (**d**) Ni60; (**e**) TiC.

For the experiment, a Q235B substrate with dimensions of 100 × 50 × 2 mm was chosen. Its surface was first rust-cleaned using sandpaper and then degreased with ethanol. The scanning electron microscope (SEM) images of Ni60, TiC and NbC are shown in Figure 1. Additionally, the particle diameter distribution diagrams of Ni60 and TiC are also presented by analyzing SEM images using Image J 1.2.5 software. Due to the extremely small particle size of NbC and its cotton-like agglomeration, the software analysis is inaccurate. The average particle diameter of Ni60 is 80.93 μm, the average particle diameter of TiC particles is 156.16 μm, and the average particle diameter of NbC is 1.85 μm (manufacturer provided). The chemical compositions of Ni60 and Q235B are detailed in Table 1.

The pre-placed powder approach was employed. A standard mold, 2 mm in thickness, was placed on the pre-conditioned substrate. The mixed powder was introduced into the mold and compressed. Subsequently, the mold was removed.

**Figure 2:**
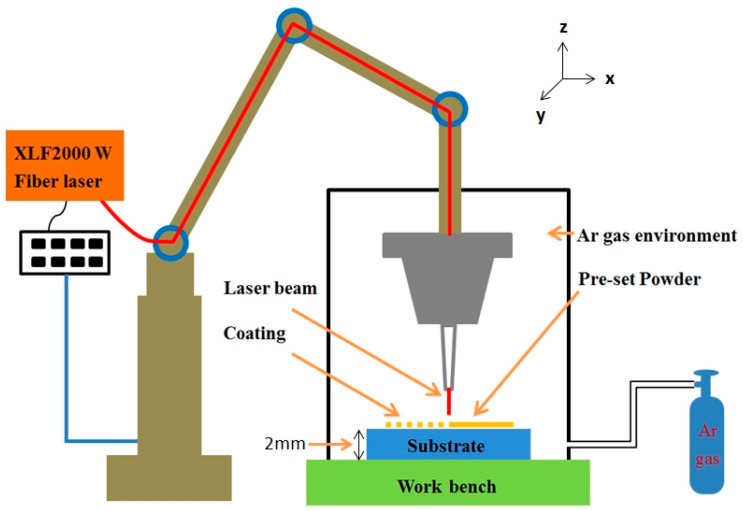
The schematic diagram of the laser processing system.

The processing was conducted by means of the XL-F2000W fiber continuous-wave (CW) laser (Gaussian beam, $M^{2}=2.8,\;\lambda =1080\;nm$) processing system (Model: XL-F2000W; Manufacturer: Shenzhen Han’s Photonics Technology Co., Ltd., Shenzhen, China). Continuous-wave (CW) lasers are more suitable for large-area laser cladding due to their stable output power and high process efficiency [32]. The processing parameters were established as detailed below: the laser power was 1200 W, the defocusing amount was +5 mm, the scanning speed was 700 mm/min, the cladding length was 40 mm, the number of passes was 12, the interval between each pass was 1.2 mm, and the shielding gas was Ar gas. The gas was ventilated initially for 20 min and then maintained in a flowing state until the conclusion of the experiment. The schematic diagram of the laser processing system is shown in Figure 2. The marking method for every sample in this experimental study is documented in Table 2.

The three samples, which were fabricated in the specified size of 10 × 10 × 2 mm, were embedded in a mold using a cold-mounting solution. Microstructural as well as elemental distribution analyses were carried out using a field-emission scanning electron microscope (FEI, QUANT 250, Eindhoven, The Netherlands) equipped with an energy-dispersive spectrometer (EDS, Noran System 7, Thermo Fisher Scientific, Waltham, MA, USA). The phase composition of the samples was detected by an X-ray diffractometer (XRD, XRD-6100, Shimadzu, Kyoto, Japan) under the conditions of 30 mA and 40 kV. Spanning from 10° to 90°, the diffraction angle was accompanied by a scanning speed set at 4°/min.

The microhardness of the sample cross-section was measured with a Vickers hardness tester, model MHVD-1000AT, manufactured by Yizong Precision Instrument Co., Ltd., Shanghai, China. The load was 20 N. The load dwell time was 10 s. Three measurements were taken in the same horizontal direction, and the average value was used as the final result to ensure the accuracy of the experimental results. The step length in the vertical position measured 0.1 mm.

To measure the friction coefficient and wear volume, a pin-disc-type friction wear tester was employed. This tester, equipped with a displacement sensor, is the SFT-2M model. It was produced by Lanzhou Zhongkehua Science and Technology Development Co., Ltd., in Lanzhou, China. The test was conducted with the following parameters: load, 30 N; rotational radius, 2 mm; rotational speed, 200 r/min; test duration, 60 min.

The three-electrode system was utilized, where the working electrode was the coating, the reference electrode was the saturated calomel electrode, and the auxiliary electrode was the platinum sheet. Electrochemical tests were performed on the samples at ambient temperature. The testing medium was a 3.5 wt% sodium chloride (NaCl) solution. An electrochemical workstation (Model: CS350M, Manufacturer: Corrtest, Wuhan, China) was employed to analyze the corrosion resistance of the samples. Electrochemical impedance spectroscopy (EIS) was carried out on the samples. The frequency range applied was from 10^−1^–10^5^ Hz. Subsequently, the measurement of the Tafel polarization curves of the samples was conducted. The scanning range was set as −0.5 V–1.5 V (vs. OCP), while the scanning rate was fixed at 0.01 V/s.

## 3. Result and Analysis

### 3.1. Metallographic Analysis

**Figure 3:**
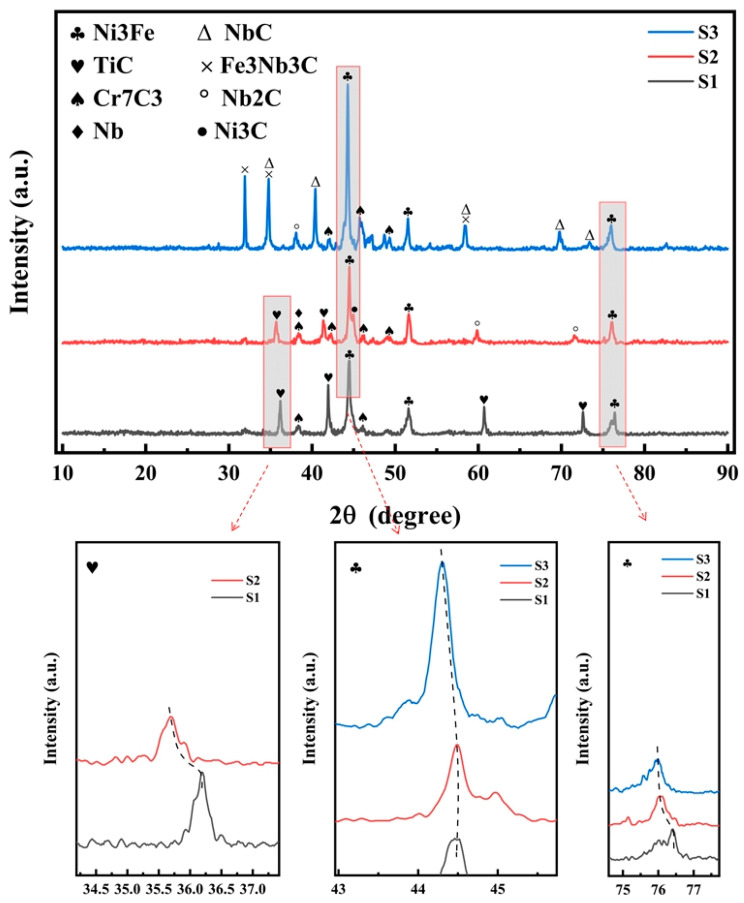
XRD spectrum of the sample.

Figure 3 illustrates the X-ray diffraction (XRD) patterns of the samples. The main peaks of the three samples are all composed of Ni_3_Fe phase peaks, which concurs with the research outcomes of Luo et al. [33]. First, observe the S1 sample. After adding 20% TiC, the TiC phase and Cr_7_C_3_ phase appear in the coating. This indicates that there are unmelted TiC particles in the S1 coating. In addition, a part of TiC is melted into Ti and C, among which C reacts with Cr in Ni60 to form the hard phase of Cr_7_C_3_. Second, observe the S2 sample. Adding 10% TiC and NbC simultaneously makes the coating generate more new phases, including Ni_3_C, Nb and Nb_2_C. After adding NbC and TiC, under the action of the laser, NbC decomposes to produce Nb and C, and TiC is decomposed into Ti and C. The free C atoms will react with Cr and Ni elements to form carbide hard phases. And a small part of the remaining free C reacts with Nb to form Nb_2_C. The reaction equations are shown in Equations (1)–(5). Finally, observe the S3 coating. After adding 20% NbC, new phases of NbC and Fe_3_Nb_3_C are generated in the coating. This shows that when the addition amount of NbC reaches 20%, the unmelted NbC phase will appear in the coating.

(1) TiC→Ti+C

(2) NbC→Nb+C

(3) $$3Ni+C\to {Ni}_{3}C$$

(4) $$7Cr+3C\to {Cr}_{7}C_{3}$$

(5) $$2Nb+C\to {Nb}_{2}C$$

Based on Bragg’s law, when there is an increase in lattice spacing, the main peak position moves to the left [34,35,36,37]. In the cooling phase of LC, the generation of substantial residual tensile stress is probable, which subsequently results in an expansion of the lattice spacing and a leftward shift in the main peak position [38]. Secondly, when larger-sized particles are added to Ni60, lattice distortion occurs, which subsequently leads to an increase in lattice spacing [39]. As shown in the partial enlarged view of Figure 3, when the addition amount of TiC gradually decreases and the addition amount of NbC gradually increases, the main peak position migrates leftward incrementally. This indicates that the introduction of NbC results in lattice distortion, which also correlates with a decrease in the grain size of the coating. Moreover, the degree of lattice distortion caused by NbC to the Ni60 coating is greater than that caused by TiC.

### 3.2. Microscopic Morphology

**Figure 4:**
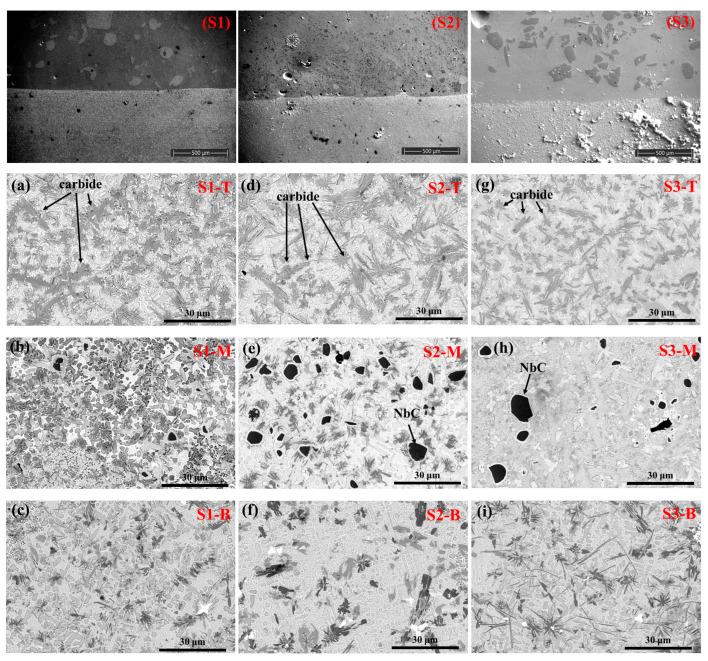
Cross-sectional SEM image of sample: (**a**–**c**) S1 sample; (**d**–**f**) S2 sample; (**g**–**i**) S3 sample.

The morphology of the samples is displayed in Figure 4, where morphological transitions in several locations are visible. The pictures in the top row are schematic diagrams of the substrate/coating interfaces of samples S1, S2, and S3. The diagrams in each column below correspond to the SEM images of the top (S1-T, S2-T, S3-T), middle (S1-M, S2-M, S3-M), and bottom (S1-B, S2-B, S3-B) regions of the coatings. The cooling rate of LC is extremely fast. According to the constitutional supercooling criterion [40,41], the crystal growth of the coating is markedly affected by the temperature gradient *G* and the solidification rate *R*. In the bottom and middle regions of the Ni60 coating, the growth of planar and cellular crystals obstructs heat dissipation in the micro-molten pool, thus reducing the G/R ratio. As solidification progresses, the growth regions of planar and cellular crystals become unstable, subsequently decomposing into smaller columnar crystals. At the top of the coating, the molten pool gradually contracts, and the interface shifts upward. The latent heat of crystallization of the liquid metal can be released into both the lower substrate and the upper air, yielding the smallest G/R ratio. Consequently, the formation of crystals without a specific orientation is favored, and the crystal size is smaller and more fragmented. After adding NbC, unmelted NbC particles appear in the middle regions of the S2 and S3 samples. It is worth noting that the hard carbide phases are present in all three coatings, a finding consistent with the XRD results. Moreover, more and more evenly distributed hard carbide phases appear in the uppermost section of the coating of the S2 sample. The hard carbide phases in the S3 are the smallest among the three coatings. In addition, compared with the carbide contents in the bottom and middle regions of the coatings, the carbides in the top regions of the three coatings are more dense. This is because during the laser cladding process, after NbC or TiC is decomposed, due to the convective effect of the laser, the C element accumulates in the top region of the coating, thus forming a denser distribution of carbides in the top portion of the coating.

### 3.3. Micro-Hardness

**Figure 5:**
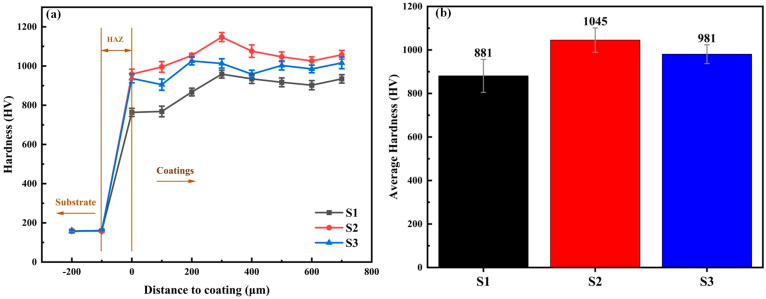
(**a**) The hardness distributions; (**b**) average microhardness.

Figure 5a shows the cross-sectional hardness distribution of the three coatings, while Figure 5b presents the calculation results of the average hardness of the S1, S2 and S3 samples. The average microhardness value of the S1 sample is 881 HV, 1045 HV for the S2 sample, and 981 HV for the S3 sample. Compared with the hardness of Ni60 without additions (672.8 HV) [33], the hardness of all three coatings is significantly improved, indicating that the addition of hard phases can indeed enhance the hardness of Ni60. The coating with 10% TiC and 10% NbC exhibits relatively higher hardness than S1 and S3. It is worth noting that adding 20% NbC results in a higher hardness value than adding 20% TiC. The main reason is that the degree of lattice distortion caused by NbC to the coating is greater than that caused by TiC, which was mentioned in the XRD analysis. The greater the degree of lattice distortion, the finer the grain size, the fewer the number of dislocations in the dislocation group, the smaller the stress concentration, and the harder the material [42,43]. In addition, through X-ray diffraction (XRD) and scanning electron microscopy (SEM) analyses, it was found that the S2 coating contains more hard carbide phases with more uniform dispersion. The carbide phases have undergone a process of redissolution and precipitation at higher temperatures, resulting in smaller sizes and a more uniform distribution. The significant increase in the density of fine carbides plays a decisive role in enhancing the hardness [44], which are important reasons for the relatively higher hardness of the S2 sample.

### 3.4. Wear Resistance

**Figure 6:**
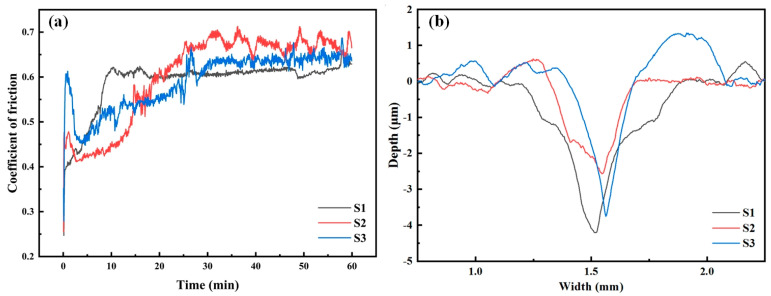
(**a**) Coefficient of friction; (**b**) two-dimensional wear profiles.

Figure 6a shows the COF curves of the three coatings when they are rubbed against the 5 mm Si_3_N_4_ steel balls. The friction coefficients of the three coatings all demonstrate pronounced initial variations, which are likely caused by the running-in of the worn surface and the sinking of the indenter [45]. First, observe sample S1. Among the three coatings, sample S1 shows the most stable COF. This indicates that adding 20% TiC can stabilize the COF curve of the Ni60 coating to a certain extent. Second, observe sample S2. The friction curve of sample S2 shows the lowest COF within the first 15 min. However, after 15 min, the COF of sample S2 gradually increases and fluctuates violently. The main reason is that, as can be found in the SEM analysis, there are the most hard carbide phases in the S2 coating. In the process of wear, when the hard carbide phases are dislodged by wear, they will wear away the coating surface together with the steel ball. This will enhance the COF of the coating and bring about fluctuations. The same situation also occurs with sample S3. But due to the smaller amount of carbides, the fluctuations are not as violent as those of sample S2.

By observing the wear profiles of each coating in Figure 6b, it can be found that the wear volume of sample S2 is the smallest, followed by that of sample S3, and then that of sample S1. Calculate the wear rate of the coating according to Equation (6) [46]:

(6) L=V/(N×d)

Among them, *L* represents the wear rate, *V* (mm^3^) denotes the wear volume, *N* (N) signifies the load, and *d* (m) indicates the total sliding distance. It can be calculated that the wear rate of S2 is the smallest, which is $1.312{\;\times \;10}^{-5}$ mm^3^N^−1^m^−1^. Next is sample S3, with a wear rate of $1.946{\;\times \;10}^{-5}$ mm^3^N^−1^m^−1^. And sample S1 has the largest wear rate, which is $1.312{\;\times \;10}^{-5}$ mm^3^N^−1^m^−1^. This indicates that sample S2 has the best wear resistance. This is consistent with the test results of hardness. Generally speaking, the hardness is directly proportional to the wear resistance of the coating.

**Figure 7:**
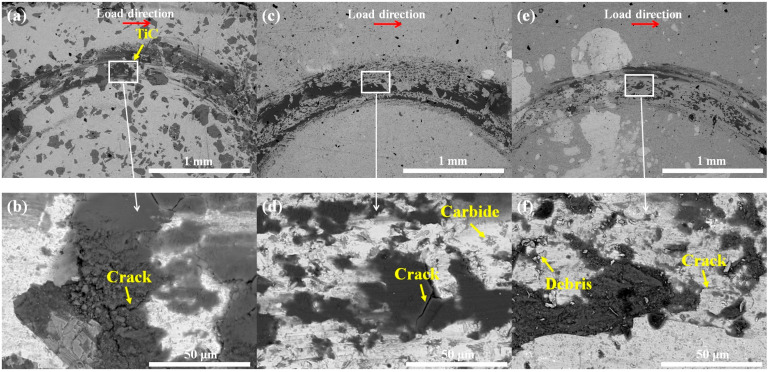
The SEM of wear trace of samples, S1 sample: (**a**,**b**); S2 sample: (**c**,**d**); S3 sample: (**e**,**f**).

Figure 7 illustrates the SEM images of the wear tracks of each sample after the wear test. The upper figures (a), (c), and (e) are contour diagrams, and the lower figures (b), (d), and (f) are the corresponding partial enlarged views. The indenter loading direction is indicated in the figures. Firstly, it is evident that the worn surface of sample S1 features some deep grooves and a modest quantity of wear debris, predominantly indicative of abrasive wear. In addition, there are many TiC particles on the coating surface, as shown in Figure 7a. The grooves of samples S2 and S3 are much shallower than those of S1, indicating that the addition of NbC particles can reduce abrasive wear. Moreover, the hard carbide phases are evenly distributed on the worn surface of sample S2. It is worth noting that only a small number of wear fragments are present within the wear profile of sample S3, which indicates that during the wear process, the carbides fall off due to wear, aggravating the wear situation. In addition, cracks are found within the wear profiles of all three samples, indicating that all three coatings exhibit fatigue wear.

### 3.5. Corrosion Resistance

**Figure 8:**
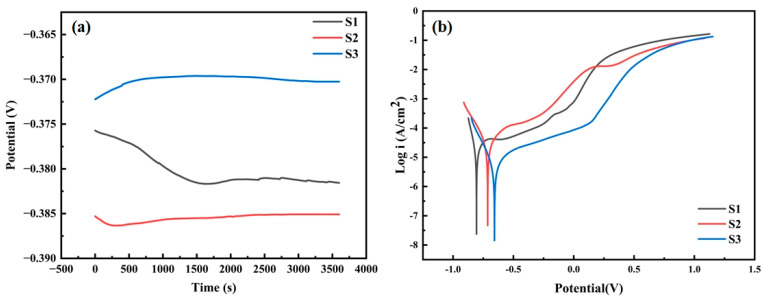
(**a**) The OCP curve of all coatings; (**b**) the Tafel polarization plot of all coatings.

Open circuit potential (OCP) tests were conducted on all samples for 1 h to determine whether a stable passive film was formed on the sample surfaces. Before the test, the samples were immersed in a 3.5% NaCl solution for 2 h. The obtained OCP curves are shown in Figure 8a. The analysis shows that the OCP of the S2 and S3 coatings tended to a stable state relatively early. This indicates that after the 2 h of immersion treatment, passive films were formed on the surfaces of the S2 and S3 coatings. In contrast, the OCP curve of the S1 coating continued to decline and only tended to be stable after 25 min. This suggests that the S2 and S3 samples are more likely to form stable passive films and have better corrosion resistance.

In addition, the final open-circuit voltages of the three samples are not significantly different. Comparatively, the steady-state open-circuit voltage of the S2 coating is the highest, indicating that it has better steady-state response characteristics and is more likely to passivate and form a stable passive film. As a result, it has a stronger anti-corrosion ability, effectively reduces the sensitivity to corrosion, and thus slows down the rate of the corrosion reaction [47,48].

The Tafel polarization curves of all the samples are depicted in Figure 8b. The anodic passivation region is a key indicator for evaluating the stability of the passivating film [47]. A wider and more stable passivation region means that there is a stable passivation film in the corrosive environment of the coating, indicating that these coatings have excellent corrosion resistance. In the anodic region, all three coatings enter the passivation region. This phenomenon indicates that it is possible for oxides and hydroxides to form on the surfaces of these three coatings, and then a passivation film is generated [48]. In the passivation region, as the voltage rises, the corrosion current of the S1 and S2 coatings remains almost constant, indicating that the surfaces of these coatings are in a stable passivation state. It is worth noting that the S3 coating has the lowest passivation current density and a larger passivation region, which highlights its strong surface passivation ability [49].

The self-corrosion potential (E_corr_) and self-corrosion current density (I_corr_) obtained by the Tafel extrapolation method are significant parameters for measuring the corrosion resistance of the coatings, with the outcomes depicted in Table 3. The E_corr_ is a concept within thermodynamics, signifying the probability of corrosion occurring; the I_corr_ is a corrosion kinetic parameter that can clearly reflect the corrosion rate [50]. The S3 coating has the largest E_corr_ and the smallest I_corr_, followed by the S2 coating. The S1 coating has the smallest self-corrosion potential (E_corr_) and the largest self-corrosion current density (I_corr_). This indicates that, from the perspective of the thermodynamic and kinetic parameters of corrosion, the S3 coating exhibits superior corrosion resistance, the S2 coating is intermediate, and the S1 coating has inferior corrosion resistance.

**Figure 9:**
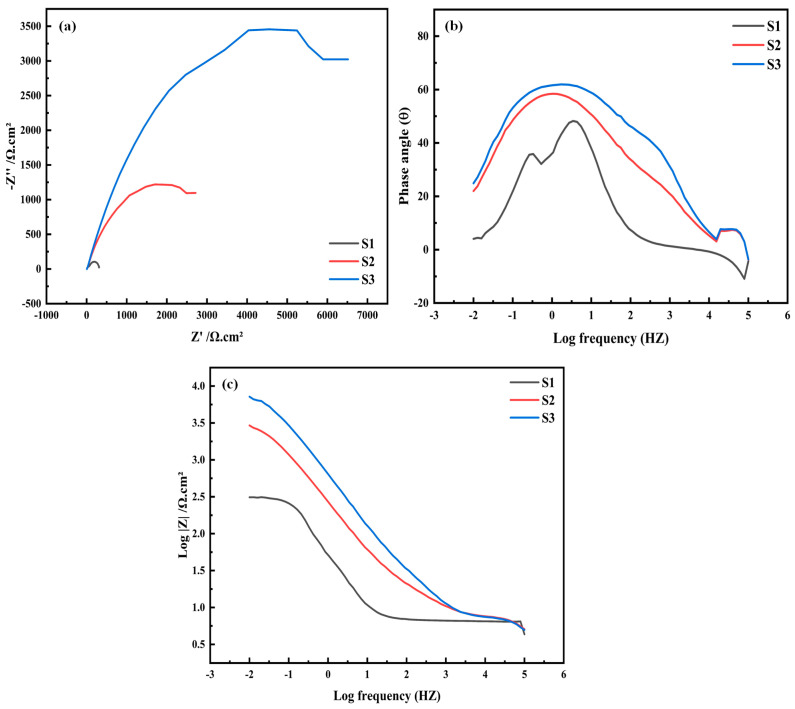
EIS spectra for coatings: (**a**) Nyquist plot; (**b**,**c**) Bode plot.

To more comprehensively investigate the properties of the surface passivation film of the three coatings and their kinetic characteristics during the corrosion process, EIS tests were conducted on all the samples. The Nyquist plot is presented in Figure 9a, where it can be seen that all samples display a nearly identical concave capacitive semi-circular shape. And this shape generally implies the existence of a charge transfer mechanism on the inhomogeneous surface, thus fully confirming the presence of the passivation film [51,52].

By analyzing the situation in the high-frequency region in depth, it can be found that the capacitance arc radii are in the following order: the capacitance arc radius of the S3 coating exceeds that of the S2 coating, while the S2 coating is greater than that of the S1 coating. The greater the capacitance loop radius, the higher the charge transfer resistance of the coating, the more challenging the electron transfer, the poorer the conductivity, the higher the impedance, and consequently, the stronger the corrosion resistance. Accordingly, among these three samples, the S3 coating demonstrates superior corrosion resistance, whereas the S1 coating exhibits the most inferior corrosion resistance.

Figure 9b,c show the Bode plots of the three coatings. Within the low-frequency range, the value of the impedance modulus can reflect the situation of the charge transfer resistance. Consequently, the overall corrosion resistance of the coatings can be intuitively evaluated through the parameter of |Z|0.01 Hz [51,52]. Through analysis, it is known that the values of |Z|0.01 Hz are in the order of: S3 coating > S2 coating > S1 coating, and the order of the magnitude of the phase angles is also the same. This order is consistent with the variation trend of the radius of the capacitive reactance arc in the Nyquist plot. The increase in the values of |Z|0.01 Hz and the phase angle indicates an increase in the charge transfer resistance, which further confirms that a thicker and denser passive film has been formed in the S3 coating. As a consequence, it notably elevates the protective efficacy of the coating.

In conclusion, the incorporation of NbC particles can significantly enhance the corrosion resistance of Ni60. The primary reason is that there is an equilibrium potential difference between the carbide phase and the Ni_3_Fe phase, leading to the formation of a corrosion microcell where the hard carbide phase acts as the cathode and the Ni_3_Fe phase serves as the anode. In accordance with the area effect of the corrosion microcell, an increase in the cathode area results in a higher anode current density, which facilitates electrochemical corrosion [53]. By observing the SEM image of the top of the coating, it can be found that the area of the hard carbide phase of the S1 sample is the largest, and the S2 sample has the most hard carbide phases. Both of these factors will increase the cathode area of the coating, thereby reducing its corrosion resistance. Moreover, studies have indicated that the incorporation of Nb can facilitate the formation of the passivation film and enhance its protective performance, thereby enhancing the corrosion resistance of the coating [54,55]. This further explains why the corrosion resistance of the coating improves as the NbC content increases.

## 4. Conclusions

In this study, laser-clad Ni60-based composite coatings reinforced with TiC and NbC particles were systematically investigated for their microstructural, mechanical, and electrochemical properties. The following key conclusions were drawn:

(1) The addition of TiC and NbC significantly altered the phase composition and microstructure of the coatings. S1 contained residual TiC particles and Cr_7_C_3_ carbides, while S2 formed Ni_3_C, Nb_2_C, and fine carbides. S3 exhibited partial dissolution of NbC, leading to the formation of Fe_3_Nb_3_C and Nb_2_C phases.
(2) Lattice distortion induced by NbC was more pronounced than that by TiC, as evidenced by XRD peak shifts, contributing to refined grain structures and enhanced mechanical properties.
(3) Due to the synergistic effect of uniform carbide distribution and severe lattice distortion, the average microhardness measured for the S2 coating is 1045 HV, slightly higher than 881 HV for S1 and 981 HV for S3, but all are significantly higher than the hardness of Ni60 (672.8 HV).
(4) Wear resistance followed the order S2 > S3 > S1, with S2 showing the lowest wear rate attributed to its superior hardness and carbide reinforcement. Abrasive wear dominated in all coatings, with fatigue cracks observed in worn surfaces.
(5) Electrochemical tests revealed that S3 exhibited the best corrosion resistance in 3.5 wt% NaCl solution, characterized by the highest corrosion potential and the lowest corrosion current density. This was attributed to the formation of a dense and stable passivation film promoted by NbC.
(6) The combination of TiC and NbC in S2 optimized hardness and wear resistance through carbide dispersion strengthening and grain refinement. However, the increased cathode area from carbide phases slightly reduced its corrosion resistance compared to S3.

Overall, the study highlights the potential of tailoring Ni60-based coatings with mixed carbide reinforcements to balance mechanical and electrochemical properties for applications in corrosive and wear-prone environments. Future work may focus on optimizing carbide ratios and processing parameters to further enhance performance.

## Figures and Tables

**Table 1:** Chemical composition of the experiment’s supplies.

Material	Chemical Composition (Mass. %)						
	C	Si	Mn	Fe	Cr	Ni	B
Q235B	0.17	0.16	0.38	Bal.	-	-	-
Ni60	0.8	4	-	15	15.5	Bal.	3.5

**Table 2:** Names of coatings with various mass fractions.

Name of Coatings	Mass Fractions/(Mass.%)
S1	Ni60 + 20%TiC
S2	Ni60 + 10%TiC + 10%NbC
S3	Ni60 + 20%NbC

**Table 3:** Electrochemical parameters derived from Tafel curves.

	Parameter	E_corr_ (V)	I_corr_ (A/cm^2^)
Sample			
S1		−0.806	1.055 × 10^−5^
S2		−0.783	2.737 × 10^−6^
S3		−0.657	1.732 × 10^−6^

## Data Availability

The original contributions presented in this study are included in the article. Further inquiries can be directed to the corresponding author.
